# Default Mode Network structural alterations in Kocher-Monro trajectory white matter transection: A 3 and 7 tesla simulation modeling approach

**DOI:** 10.1371/journal.pone.0224598

**Published:** 2019-11-07

**Authors:** Saül Pascual-Diaz, Jose Pineda, Laura Serra, Federico Varriano, Alberto Prats-Galino

**Affiliations:** Laboratory of Surgical Neuroanatomy, University of Barcelona, Barcelona, Spain; University of Texas at Austin, UNITED STATES

## Abstract

The Kocher-Monro trajectory to the cerebral ventricular system represents one of the most common surgical procedures in the field of neurosurgery. Several studies have analyzed the specific white matter disruption produced during this intervention, which has no reported adverse neurological outcomes. In this study, a graph-theoretical approach was applied to quantify the structural alterations in whole-brain level connectivity. To this end, 132 subjects were randomly selected from the Human Connectome Project dataset and used to create 3 independent 44 subjects groups. Two of the groups underwent a simulated left/right Kocher-Monro trajectory and the third was kept as a control group. For the right Kocher-Monro approach, the nodal analysis revealed decreased strength in the anterior cingulate gyrus of the transected hemisphere. The network-based statistic analysis revealed a set of right lateralized subnetworks with decreased connectivity strength that is consistent with a subset of the Default Mode Network, Salience Network, and Cingulo-Opercular Network. These findings could allow for a better understanding of structural alterations caused by Kocher-Monro approaches that could reveal previously undetected clinical alterations and inform the process of designing safer and less invasive cerebral ventricular approaches.

## 1 Introduction

Accessing the cerebral ventricular system is a common procedure in current neurosurgery: external ventricular drainages (EVD), ventriculoperitoneal shunts (VPS) and neuroendoscopic procedures are carried out daily [1]. An EVD or a VPS can be indicated to treat hydrocephalus—the accumulation of brain fluid within the intracranial cavity—and evacuation of intraventricular blood. Without relieve, excess cerebrospinal fluid (CSF) can increase intracranial pressure resulting in herniation or other complications. Although the placement of an EVD is considered safe and it is associated with patient survival, no consensus has been reached on its impact on neurological outcomes [2]. Many authors addressing ventriculostomies focus on the accuracy of the technique and its rates of hemorrhagic and infectious complications [3]. Some authors have compared the free-hand technique accuracy with neuronavigator-assisted procedures [4, 5], while others have developed special devices to improve the rate of success [6, 7]. The frontal approach with the Kocher-Monro (KM) trajectory, which starts at Kocher’s point and ends at the foramen of Monro, seems to be one of the most common options and has become a standard approach to access the ventricular system [8]. A few connectivity studies have analyzed the damage produced to the white matter (WM) tracts which are inevitably transected along the KM trajectory [9, 10].

Previous studies of damage derived from KM ventriculostomies have consistently reported disruptions to the *genu* of the *corpus callosum* and the cingulate tract [9, 11]. Nevertheless, these studies were directed by an *a priori* selection of the tracts to be studied and, to the best of our knowledge, no global network-based studies addressing the distributed damage has been reported. From a network-based point of view, the aforementioned reported alterations in KM ventriculostomies affect critical edges of the Default Mode Network (DMN) which is a functional network structurally defined by cortical nodes whose BOLD signals are correlated during internally-oriented mental processes and lose correlation during the execution of attention-demanding tasks [12].

Structural connectivity-based approaches allow to obtain brain topology information from magnetic resonance imaging (MRI) by performing analyses based on the mathematical framework of graph theory. Connectivity matrices are constructed using a set of nodes, representing Regions Of Interest (ROI) of the brain, and a collection of edges describing the weight between them. Fundamental organizational properties in a specific sample can be compared by using a consistent set of graph-theoretical indices and network-based statistics (NBS) [13, 14]. The number of neurons in the human brain is in the order of 10^10^, forming up to 10^13^ synaptic connections. To this day, building a complete connectivity matrix accounting for every neuron (i.e. a connectome) remains an unfeasible task, given the methodological limitations faced in such high complexity systems. Nonetheless, a macroscale approach where the connectivity matrices are defined as point-to-point spatial connectivity matrices of neural pathways derived from diffusion connectivity reconstructions can be used to study the structural connectivity in the human brain [15, 16]. The study of the brain as a connectivity matrix can be used to contrast measures and check whether the resulting matrices describe (totally or partially) well known large-scale networks (e.g. Default Mode or Salience networks) [17].

In the present study, our hypotheses are: 1) The simulated KM trajectory will not produce a total disconnection between any ROI pair, 2) The simulated KM trajectory will produce a statistically significant decrease in the number of streamlines (NOS) projecting to/from arbitrary ROIs and 3) Specifically, the simulated KM trajectory will produce a statistically significant decrease in the NOS constituting white matter pathways that anatomically constrain the DMN.

## 2 Materials and methods

### 2.1 Human Connectome Project data

We performed our study on neuroimaging data from 132 randomly selected subjects from the WU-Minn 7 tesla Human Connectome Project (HCP) 1200 MRI open access dataset (http://humanconnectomeproject.org). Three and 7 tesla diffusion weighted sequences were used. For structural images, 3 tesla T1 sequences were used as 7 tesla T1 images are not available in the HCP sample.

The 3 tesla HCP acquisition was conducted on a Siemens Magnetom Skyra MRI machine. Structural imaging consisted of a T1 weighted 3D MPRAGE sequence with 224 × 244 mm of FOV with a voxel size of 0.7 mm isotropic (TR = 2400 ms, TE = 2.14 ms, TI = 1000 ms, flip angle of 8 degrees, BW = 210 HZ/Px, iPAT of 2 and the acquisition time was 7 minutes and 40 seconds) [18]. The diffusion weighted HCP acquisition parameters are summarized in Table 1 [19, 20].

**Table 1:** HCP diffusion protocols.

	HCP 3T	HCP 7T
Spatial resolution	1.25 *mm*^3^	1.05 *mm*^3^
	LR/RL phase encoding (PE)	AP/PA phase encoding (PE)
Acceleration	Multiband = 3	Multiband = 2
		GRAPPA = 3
	Partial Fourier = 6/8	Partial Fourier = 6/8
Total echo train length	84.24 msec	41 msec
Gradient strength (max)	100 mT/m	70 mT/m
*b*−values(*s*/*mm*^2^)	1000, 2000, 3000	1000, 2000
Q-space directions	270 × 2	130 × 2

Summary of main features of the HCP 3T and 7T dMRI protocols.

According to the HCP consortium, the subjects were healthy individuals that gave written consent to share anonymized brain imaging and behavioral data, and lacked a significant history of psychiatric or neurological disorders, among other conditions [21]. The inclusion criteria for this study were the availability of 3T and 7T diffusion data, Freesurfer parcellation data [22] and T1 tissue segmentation images showing sufficient quality. To the best of our knowledge, there are no reports of variables such as handedness or general intelligence affecting the neurological outcomes of KM ventriculostomies, and thus subjects were selected by simple random sampling in order to minimize bias and preserve the variance of the global dataset.

This study was conducted with the approval of the Bioethics Committee of the University of Barcelona, Institutional Review Board (IRB00003099).

### 2.2 Experimental design

We conducted our study in three different stages addressing each respective hypothesis: the first stage consisted in a graph-theoretical indices comparison from unweighted, undirected connectivity matrices (adjacency matrices); the second stage consisted in a nodal analysis from weighted, undirected connectivity matrices comparing the total number of projections for each ROI testing if the simulated KM trajectory decreases the NOS projecting to/from different ROIs; the third stage consisted in a network-based analysis to find whether the alterations, if present, comprise isolated node pairs or define specific subnetworks.

Three independent groups were created by randomly selecting 44 subjects per group (132 subjects total). Subjects from two of the groups received the simulated KM trajectory on the left and right hemispheres respectively for each field, while the third group was kept as control. The groups that received the simulated KM trajectory will be referred as virtual patients (Table 2). Given that 3T and 7T acquisitions have complementary features forming a trade-off [20], we decided to perform the study in both fields and keep the common results while discarding results found in one but not the other, as using them together can be a useful strategy for combining their benefits (i.e. 3T angular resolution and 7T spatial resolution) and reduce alpha error.

**Table 2:** Description of the groups.

	N	Age ± SD (years)	Sex (male ratio)
Global sample	132	29.50 ± 3.35	0.38
Controls	44	29.52 ± 3.71	0.36
Left transected virtual patients	44	29.34 ± 3.06	0.41
Right transected virtual patients	44	29.64 ± 3.24	0.36

Demographic description for global and groups data.

### 2.3 Kocher-Monro trajectory simulation

A virtual instrument placed along the KM trajectory was simulated. The trajectory begins at Kocher’s point and ends at the entrance of the foramen of Monro, which communicates the lateral ventricles and the third ventricle of the brain. Even though some authors propose different definitions of Kocher’s point, there is broad consensus on a point located 10 cm posterior to the nasion and 3 cm lateral to the midline, approximately on the mid-pupillary line [23]. To minimize alpha error, the diameter of the simulated instrument was set to a conservative value of 4 mm, even though broader instruments are often used in regular practice.

**Fig 1:**
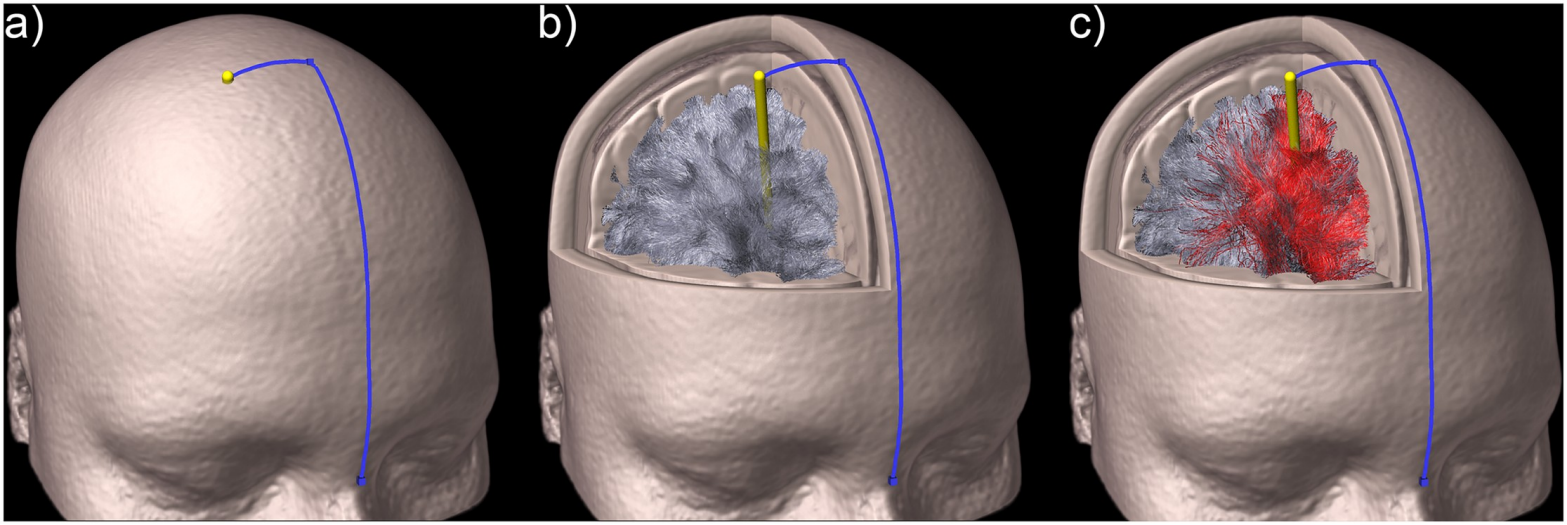
3D render of the Kocher trajectory and virtual instrument. a) Kocher’s point definition method, b) virtual instrument transecting a whole-brain tractographic reconstruction and c) virtual instrument transecting a whole-brain tractographic reconstruction showing in red the transected streamlines.

Left and right hemisphere virtual instruments were generated for each individual subject using Python [24]. A Python algorithm was used to determine the entrance point automatically after an expert anatomist determined the nasion and the foramen of Monro points in the T1 image (Fig 1). All simulations were checked by an expert anatomist after each virtual instrument was placed in the KM trajectory. A probabilistic map of the KM virtual instruments can be found in S1 File.

Fig 1 was created using Amira software 6.3 (Thermo Fisher Scientific, USA). The face of the depicted subject was substituted with the T1w template from the Montreal Neurological Institute (MNI) for illustration purpose only.

### 2.4 Tractogram reconstructions

For each individual subject and field, whole brain tractography reconstructions were performed with Mrtrix3 software [25] using the iFOD2 algorithm firing randomly distributed seeds in the WM until 10 million streamlines were obtained [26]. Tissue information provided by the Anatomically-Constrained Tractography (ACT) framework was used to provide additional information to the tracking algorithm and Multi-Shell, Multi-Tissue Constrained Spherical Deconvolution (MSMT-CSD) was performed. A maximum length limit of 200mm was defined to reduce the presence of false positives. For the virtual patient groups, the KM-induced tissular disconnections were simulated by removing streamlines transecting the virtual instrument.

### 2.5 Connectivity matrix construction

**Fig 2:**
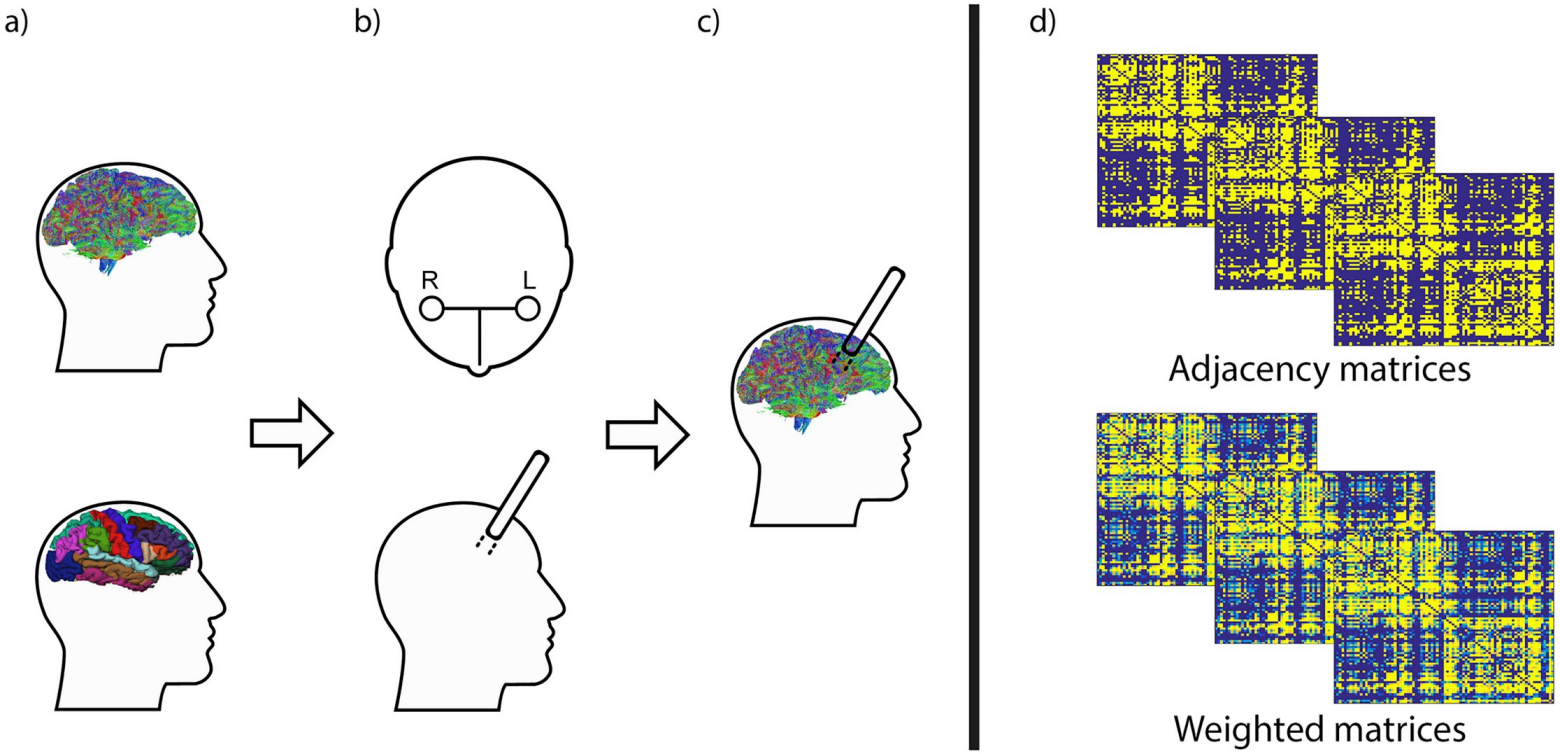
Schematic illustration of the pipeline used to obtain the connectivity matrices. a) Cortical parcellations from the Desikan Atlas and whole-brain tractography calculated for each individual subject. b) The simulated KM trajectory for each virual patient. c) Virtual instrument placement in the whole-brain tractography. d) Adjacency and weighted matrices from each group of subjects.

A graph representation was reconstructed for each subject brain connectivity network. The graphs consisted of grey matter nodes *γ* = {*γ*_*i*_|*i* ∈ 1, …, *n*} with *n* being 84 cortical areas, reflecting the parcellation of the Desikan atlas [27] in native space. Reconstructed WM connections between these areas were considered as edges *ϵ* = {*e*_*ij*_|(*γ*_*i*_, *γ*_*j*_) ∈ *γ* × *γ*} of the graph *G* = (*γ*, *ϵ*) (Fig 2). The weight between nodes was defined using Track-Weighted Fractional Anisotropy (TW-FA) imaging [28, 29]. In the tract-weighted imaging approach an image is computed based on properties of the streamlines themselves (e.g. based on the NOS in each voxel) or combining the tractrogram information using the values of an associated diffusion MRI data. Each streamline can be assigned a “weighting” corresponding to the average FA value along the streamline coordinates. The TW-FA framework can be considered as a track-informed version of the corresponding FA map which results in a directionally-informed smoothing of the tractograms, improving the graph-theoretical measures compared to using NOS matrices alone [30]. Network connections were included for node pairs present in more than half of the sample of both the controls and the virtual patients groups. [31, 32].

### 2.6 Weighted matrices resampling

Edge weights obtained from TW-FA are not normally distributed [33]. For this reason, all matrices were resampled into a Gaussian distribution by replacing values from the sorted list of TW-FA with values from a sorted list of normally distributed random values [34, 35]. Given N raw TW-FA values:

$$\begin{array}{c} x_{1},x_{2},x_{3},\ldots ,x_{n} \end{array}$$

N random samples were generated from a unit Gaussian distribution:

$$\begin{array}{c} r_{1},r_{2},r_{3},\ldots ,r_{n} \end{array}$$

The smallest raw data value was replaced with the smallest randomly generated unit Gaussian distribution value. This procedure was iterated until all raw data was replaced. The resulting normalized matrices can be used to perform inter-subject comparisons.

### 2.7 Graph-theoretical indices

The graph-theoretical indices included in this study were calculated from adjacency matrices, where each connection is only counted once: (i) clustering coefficient as a scalar value that quantifies node segregation, (ii) characteristic path length as the average number of steps along the shortest paths between connected nodes; (iii) weighted global efficiency as a measure of how efficiently the network exchanges information; and (iv) assortativity coefficient as a measure of robustness of all nodes on two opposite ends of a link where if a network’s assortativity coefficient is negative, a hub tends to be connected to non-hubs. Nodal analyses were calculated from weighted undirected connectivity matrices using the nodal strength as a measure to evaluate the number of projections for each ROI [36]. Additional description for the graph-theoretical calculations can be found at S1 Text.

All measures were computed on the individual structural normalized matrices using the Brain Connectivity Toolbox 1.2 package (BCT; https://sites.google.com/site/bctnet/) under Python.

### 2.8 Connectivity matrices statistical analysis

Graph-theoretical indices comparisons between controls and virtual patients were performed using permutation-based tests (10K permutations). The resulting *p* − *values* were corrected for multiple comparisons using false discovery rate (FDR) correction if applicable.

To check for node-level differences, a t-test for each node strength value was performed comparing controls and virtual patients after performing a Shapiro-Wilk test to confirm normality and an F test to prove homoscedasticity. The resulting values were corrected for multiple comparisons using FDR-correction if applicable.

Edge-level group differences were analyzed using the NBS framework. The NBS is a nonparametric statistical method used to tackle the multiple comparisons problem. When conducting edges comparison, the NBS allows to control the family-wise error rate (FWER), in the weak sense, when performing mass univariate hypothesis testing [37]. The NBS comprises four steps: the first step is comparing the NOS between groups by performing a two-sample t-test for each individual edge; the second step is applying a principal component threshold with an uncorrected alpha of 0.001 to form a set of suprathreshold edges; the third step is applying a permutation test to an adjacency matrix defined by the suprathreshold edges to calculate the p-values for every component; and the last step is iterating the aforementioned steps 5K times, randomly permutating selected members of the two populations each time and storing only the largest component for each iteration. The result controls the FWER at cluster level for p-values less than 0.05. To reduce the number of comparisons, only connected nodes were tested.

All statistical tests were performed using Python. The null hypothesis was rejected for corrected p-values under our significance threshold established at *α* = 0.05.

## 3 Results

### 3.1 Graph-theoretical indices analysis

**Fig 3:**
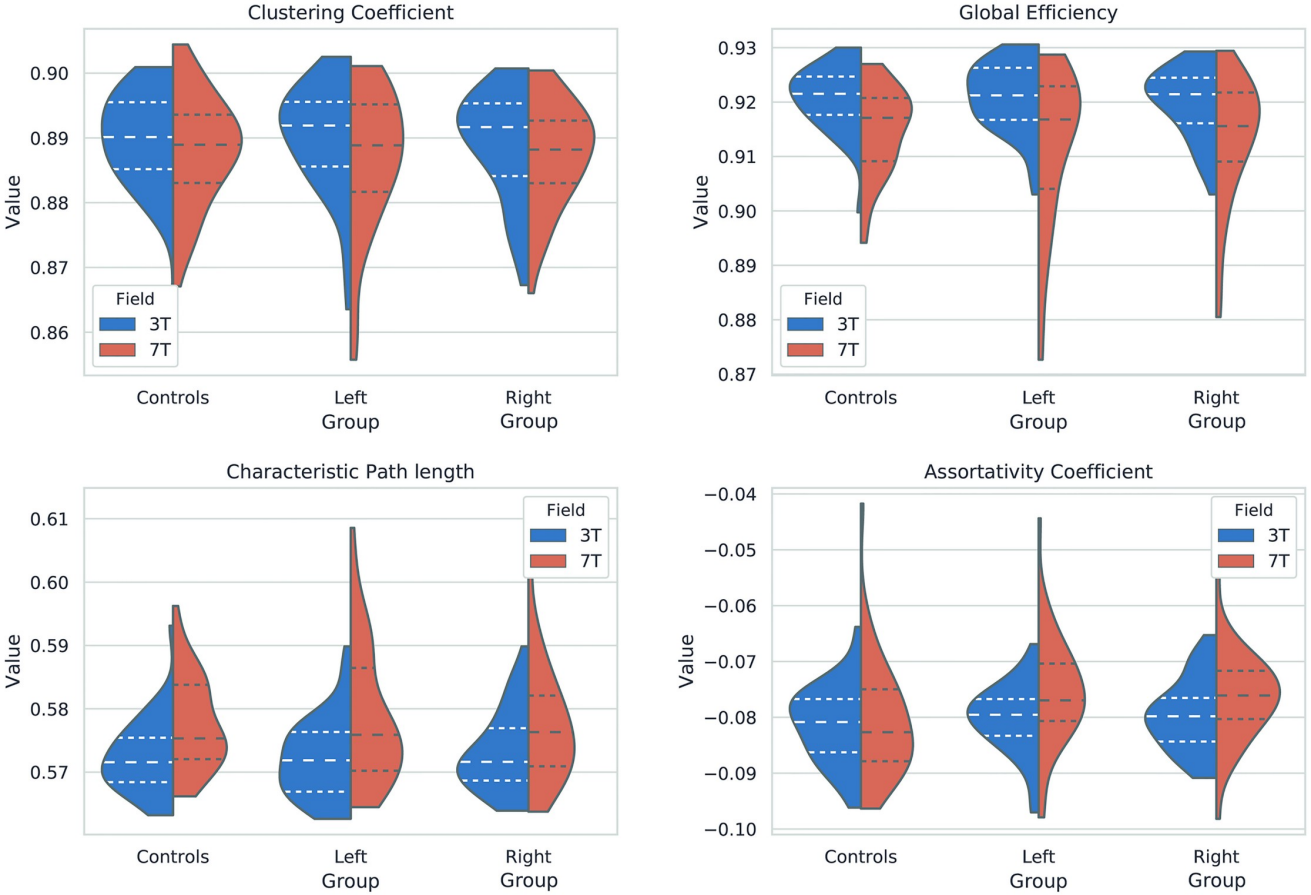
Graph-theoretical indices distributions. Violin plots showing the normalized values for the 3T and the 7T analyses for the a) clustering coefficient, b) global effiency, c) characteristic path length and d) assortativity coefficient. The horizontal dashed lines indicate the quartiles.

We have not found significant differences in any graph-theoretical index for the 3T acquisition (Table 3) nor the 7T acquisition (Table 4). Fig 3 shows the distribution of the mean values for each of the graph-theoretical indices compared for both fields.

**Table 3:** 3T Graph-theoretical indices results.

	Control	Left KM trajectory		Right KM trajectory	
	Mean ± SD	Mean ± SD	*p*	Mean ± SD	*p*
Clustering coefficient	0.890 ± 0.008	0.890 ± 0.008	0.837	0.887 ± 0.008	0.657
Characteristic path length	0.578 ± 0.007	0.578 ± 0.010	0.850	0.579 ± 0.010	0.519
Global efficiency	0.915 ± 0.008	0.912 ± 0.014	0.852	0.913 ± 0.011	0.518
Assortment	-0.080 ± 0.010	-0.076 ± 0.010	0.525	-0.076 ± 0.007	0.270

Graph-theoretical indices mean values obtained from adjacency connectivity matrices from the 3T acquisition.

**Table 4:** 7T Graph-theoretical indices results.

	Control	Left KM trajectory		Right KM trajectory	
	Mean ± SD	Mean ± SD	*p*	Mean ± SD	*p*
Clustering coefficient	0.888 ± 0.009	0.888 ± 0.010	0.574	0.887 ± 0.008	0.757
Characteristic path length	0.578 ± 0.007	0.578 ± 0.010	0.663	0.579 ± 0.010	0.588
Global efficiency	0.915 ± 0.008	0.912 ± 0.014	0.257	0.913 ± 0.011	0.406
Assortment	-0.081 ± 0.010	-0.076 ± 0.001	0.26^†^	-0.076 ± 0.008	0.26^†^

Graph-theoretical indices mean values obtained from adjacency connectivity matrices from the 7T acquisition.

^†^ FDR-corrected *p* values.

### 3.2 Nodal analysis

For nodal weighted measures, we found a significant node strength decrease in the right caudal anterior cingulate (*p* < 0.001, FDR-corrected) and the rostral anterior cingulate node (*p* < 0.001, FDR-corrected) for the right transected group. No significant results were found for the left transected group.

### 3.3 Network-based analysis

**Fig 4:**
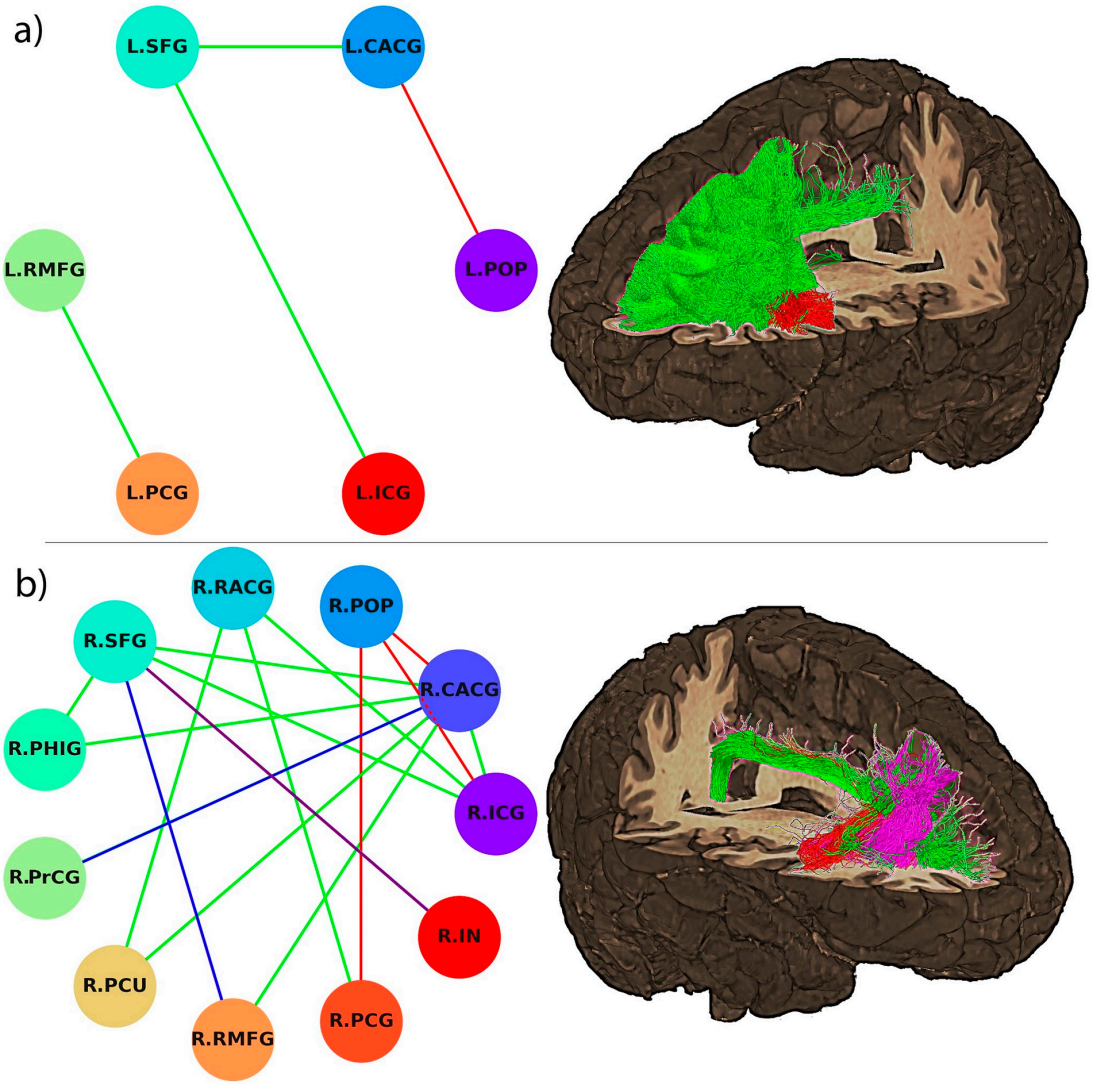
Network-based analysis results. Connectogrames representing the significant subnetworks for the network-based analyses for the a) left simulated KM trajectory and b) right simulated KM trajectory. The edges and tractograms were color-coded according to the specific subnetwork they belong to: green for the Default Mode Network, red for the Salience Network, purple for the Operculo-Cingular Network and blue for other association tracts. Node acronyms definitions: left superior frontal gyrus (L.SFG), left caudal anterior cingulate gyrus (L.CACG), left posterior cingulate gyrus (L.PCG), isthmus cingulate gyrus (L.ICG), left *pars opercularis* (L.POP), left rostral middle frontal gyrus (L.RMFG), right rostral anterior cingulate (R.RACG), right *pars opercularis* (R.POP), right caudal anterior cingulate gyrus (R.CACG), right isthsmus cingulate gyrus (R.ICG), right insula (R.IN), right posterior cingulate gyrus (R.PCG), right rostral middle frontal gyrus (R.RMFG), right precuneus (R.PCU), right precentral gyrus (R.PrCG), right parahippocampal gyrus (R.PHIG) and right superior frontal gyrus (R.SFG). The 3D renders show the streamlines of a single representative subject for illustration purpose.

Network-based analysis revealed a set of subnetworks composed of significantly (*p* < 0.05, FWER-corrected) altered WM pathways present in both 3T and 7T analyses as shown in Fig 4.

## 4 Discussion

**Fig 5:**
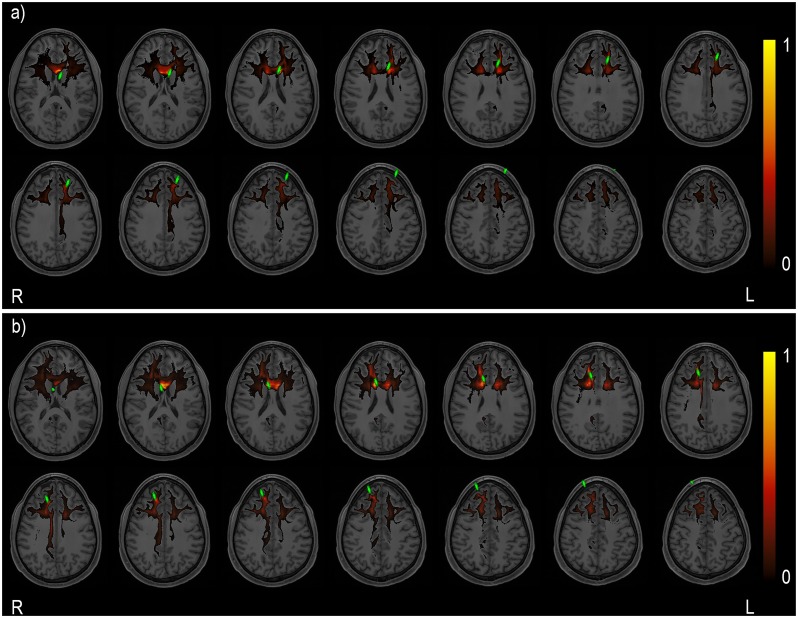
Probabilistic map for the streamlines intersecting the virtual instrument. Colormap representing the normalized probabilistic map for the streamlines transecting the virtual instrument for the a) left simulated KM trajectory and b) right simulated KM trajectory groups. Virtual instrument represented in green.

This study addresses the effects of a simulated KM trajectory in the anatomical pathways that are present in the KM transected region, without restricting our scope to a specific *a priori* selected network. Given that tractography reconstruction usually shows a number of false positive streamlines [33], the insertion of the virtual instrument produced a diffuse effect (Fig 5) that cannot be trivially attributed to any specific pathway, but can be approached from a graph-theoretical perspective. Furthermore, the graph theoretical parameters derived from NOS-based connectivity matrices are robust against weak connections pruning [38] while remaining sensitive to alterations in strong pathways, making them an ideal choice to identify significantly altered pathways as a result of the simulated KM trajectory. The consistency and the reproducibility of the presented methodology for the KM trajectory allowed us to perform our study on a large-scale simulation in an automated manner.

There were two possible field strengths to consider when conducting a study using the HCP dataset. Given the relatively small literature on 7T MRI research and the widespread opinion that 7T is disadvantageous for diffusion imaging, the 3T acquisition was a major consideration for this study. In the HCP sample, the two protocols are comparable in terms of image quality and SNR for matched b-values images. This can be explained by the HCP 3T acquisition having a greater angular resolution, and the HCP 7T acquisition having a shorter TE (71ms vs 89ms), a longer TR (5.5s vs 7s) and a greater spatial resolution (1.05mm vs 1.25mm) which results in reduced partial volume effect [39]. The higher spatial resolution of the HCP 7T data allows for a more precise tractographic reconstruction of long range pathways constituting the large-scale networks when combined with the 3T data [19]. For this reason, the methodology in the present study aims to combine their benefits resulting in a conservative approach to control alpha error, which is preferable in a simulated study.

The graph-theoretical indices group comparison between virtual patients and controls did not show topological differences produced by the virtual instrument. Thus, we cannot conclude that the KM trajectories had produced a total disconnection between any ROI pair. This is in accordance with hypothesis 1 and with the lack of significant neurological outcomes reported in the literature. Nonetheless, this negative result is not enough to rule out possible alterations that can be detected by local methods.

When approaching the foramen of Monro, the virtual instrument traverses the anterior region of the Anterior Cingulate Cortex (ACC). The nodal analysis revealed a strength decrease for the right caudal anterior cingulate and the rostral anterior cingulate cortices for the right transected group, which are precisely the nodes that form the ACC in the Desikan parcellation. The *cingulum* is formed by a densely packed anteroposterior bundle whose width is comparable with the virtual instrument’s width, making it likely for the transection to produce a significant (altough not complete) disconnection, compared to other connections such as the commisural callosal fibers which present a broader fan-like shape and are thus less affected by the virtual instrument transection.

The ACC is the main anterior node of the DMN and shows coordinated activity under task-free conditions with the posterior cingulate cortex (PCC), angular gyrus, middle and superior frontal gyri, and middle temporal gyrus [12]. This functional network can be seen as an interplay of functionally and anatomically dissociable dorsal attention and frontoparietal control networks reflecting competition between exogenous and endogenous loci of information processing [40]. Furthermore, the DMN has also been studied from a strictly structural approach [41]. The network analysis revealed an asymmetric set of subnetworks with decreased structural connectivity depending on the hemisphere intervened, compatible with the DMN. The affected subnetworks were right lateralized and consisted of connections between the PCC, SFG, and the lateral occipital/parietal cortices (LOC/LPC). The streamlines constituting these WM pathways include portions of the superior and inferior longitudinal fasciculi, *cingulum*, and the *genu* of the *corpus callosum*. These results are consistent with the nodes and the edges reported elsewhere in DMN connectivity matrices, in structural diffusion MRI acquisitions, and when performing resting state tasks during functional MRI acquisitions [42, 43]. The DMN can be separated in different core nodes: the ventral medial prefrontal cortex (VMPC), the dorsal medial prefrontal cortex (DMPC), the PPC and the retrosplenial cortex, the lateral temporal cortex; and the precuneus plus the lateral parietal cortex [44]. We found statistically significant decrease in node strength connecting the PCC with the DMPC, the PCC with the VMPC; and connections between the precuneus and the DMPC. Our results suggest that access to the cerebral ventricular system through the KM trajectory produced a statistically significant decrease in the normalized NOS constituting main WM edges that anatomically constrain the DMN in accordance with hypothesis 3.

The ACC also has anatomical connections with the nodes forming the Salience Network (SN), which is a functionally defined large scale network that supports the detection of emotionally salient stimuli, and it is anticorrelated with the DMN [45]. This network has large nodes connecting the anterior insula and the ACC and distinct limbic areas including the amygdala, ventral striatum, thalamus, hypothalamus and substantia nigra/ventral [46]. For the right transected group, we found significant differences in the connection strength between the insula and the dorsolateral prefrontal cortex, affecting the cortical subnetwork of the SN.

The NBS also revealed a significant connection strength decrease in the pathways connecting the different subcomponents of the ACC with the *pars opercularis* for both left and right transected groups. Connections between these ROI pairs are described as the main edges of the cingulo-opercular network (CON), which is involved in general attentional control, and is composed of ACC, *pars opercularis*, insula and thalamus nodes [47]. This analysis also detected differences in a set of nodes comprising short association fibers connecting the right superior frontal gyrus and the right rostral middle frontal gyrus that were directly affected by the virtual instrument trajectory and do not play a direct role in large scale brain networks.

The simulated KM trajectory is a symmetrical approach to access the ventricular system and thus we should expect symmetrical results for the NBS analysis in the case of a perfectly symmetrical brain and frontal bone. Nonetheless, a global anterior-posterior “torque” pattern is known to induce a left-right cortical asymmetry that is a feature of the human brain’s structure and function. The HCP consortium reported left-right asymmetries characterizing the structural and functional connectivity in perisylvian language cortical regions in the HCP dataset [48]. In accoradnce with this evidence, we found left-right differences in *pars operacularis*, rostral anterior cingulate, caudal middle frontal, isthmus cingulate, precuneus, rostral middle frontal and superior frontal nodes, whose cortical structure lateralization has also been reported [49].

Given that the DMN, the SN and the CON are defined in terms of temporally correlated BOLD signal between ROI pairs, caution should be exercised when reaching conclusions about the functional aspects of these networks solely from structural evidence; thus, that is not the objective of the present work. The structural study of the WM pathways that constrain the significant networks found in this work can be used to better understand the anatomical scaffold of these functional networks.

### 4.1 Limitations

Studies based on structural connectivity matrices have inherent limitations [50]. Fiber tracking is an indirect approximation to actual structural connectivity, it has known biases [33, 51] leading to a limited relationship to the underlying anatomy [52] and has limitations reconstructing long-range connections [53].

The simulated KM trajectory was performed by removing the streamlines transecting the virtual instrument to reproduce a mechanical disconnection. All the statistical analyses were performed using normalized NOS-based metrics as it is unclear how to simulate the effects of the streamline removal in other diffusion metrics such as the fractional anisotropy or the mean diffusivity. Furthermore, this approach restricts the interpretation of this study to a purely structural framework, as translating the simulated disconnections to other MRI modalities is a non trivial and open question.

This study was conducted among a sample of healthy subjects in which we simulated a 4-mm wide KM disruption. In the regular practice broader instruments are also used and several punctures are often needed to access the ventricular system [54]. For these reasons, greater WM damage than the estimated in this study could be produced during a regular ventriculostomy. KM trajectory is often used for major cause in cases of hydrocephalia, stroke and brain injury, where the brain structure is already altered, and thus could produce asymmetric disruption to the brain connectivity affecting the structural and functional connectome in heterogeneous ways that are beyond the scope of a simulation study. Furthermore, the catheter placed along the KM trajectory could displace or deform the WM. Connectomes from virtual patients may be useful as a first approach but does not necessarily reflect the brain organization for real patients.

### 4.2 Future work

Future studies should be conducted to assess how the KM trajectory affects the DMN, SN and CON including functional information. This could be approached by delimiting the corresponding networks ROIs from the fMRI dataset that is available in the HCP sample.

An fMRI study with patients who have undergone a real ventriculostomy using the statistical analysis based on graph-theoretical indices and NBS would be necessary to validate the results reported in this work. A graph-theoretical apprach may be used to analyse signal acquisitions from patients by using other modalities such as MEG to correlate these results with the potential clinical outcomes derived from KM interventions.

### Conclusions

We performed a simulation study of the structural connectivity alterations produced by the KM ventriculostomy. By comparing structural brain networks between virtual patients and healthy controls and applying network-based analyses, we found a right lateralized subnetwork in the virtual patients group that is consistent with a subset of the DMN, SN and CON. The findings in the present study could allow for a better understanding of structural alterations after KM approaches that could reveal previously undetected clinical alterations and inform the process of designing safer and less invasive cerebral ventricular approaches.

## Supporting information

S1 File Summarized map for virtual instruments placement. Probabilistic map showing the spatial trajectory followed by the Kocher Monro virtual instruments used to perform the simulations.
(GZ) Click here for additional data file.

S1 Text Mathematical expressions of graph-theoretical indices used to describe brain topology used in this study. (TEX)

## References

[1] Muralidharan R. External ventricular drains: Management and complications. Surgical neurology international. 2015;6(Suppl 6):S271–4. 10.4103/2152-7806.157620 26069848 PMC4450504

[2] Lovasik BP, McCracken DJ, McCracken CE, McDougal ME, Frerich JM, Samuels OB, et al The Effect of External Ventricular Drain Use in Intracerebral Hemorrhage. World neurosurgery. 2016;94:309–318. 10.1016/j.wneu.2016.07.022 27436212

[3] Roitberg BZ, Khan N, Alp MS, Hersonskey T, Charbel FT, Ausman JI. Bedside external ventricular drain placement for the treatment of acute hydrocephalus. British journal of neurosurgery. 2001;15(4):324–327. 10.1080/02688690120072478 11599448

[4] Abdoh MG, Bekaert O, Hodel J, Diarra SM, Le Guerinel C, Nseir R, et al Accuracy of external ventricular drainage catheter placement. Acta neurochirurgica. 2012;154(1):153–9. 10.1007/s00701-011-1136-9 21892637

[5] Kakarla UK, Kim LJ, Chang SW, Theodore N, Spetzler RF. Safety and accuracy of bedside external ventricular drain placement. Neurosurgery. 2008;63(1 Suppl 1):ONS162–6; discussion ONS166–7. 10.1227/01.neu.0000335031.23521.d0 18728595

[6] Ghajar JB. A guide for ventricular catheter placement. Technical note. Journal of neurosurgery. 1985;63(6):985–6. 10.3171/jns.1985.63.6.0985 4056916

[7] Schaumann A, Thomale UW. Guided Application of Ventricular Catheters (GAVCA)–multicentre study to compare the ventricular catheter position after use of a catheter guide versus freehand application: study protocol for a randomised trail. Trials. 2013;14(1):428 10.1186/1745-6215-14-428 24330776 PMC3866392

[8] Srinivasan VM, O’Neill BR, Jho D, Whiting DM, Oh MY. The history of external ventricular drainage. Journal of neurosurgery. 2014;120(1):228–36. 10.3171/2013.6.JNS121577 23889138

[9] García S, Rincon-Torroella J, Benet A, Oleaga L, González Sánchez JJ. Assessment of White Matter Transgression During Neuroendoscopic Procedures Using Diffusion Tensor Image Fiber Tracking. World neurosurgery. 2017;99:232–240. 10.1016/j.wneu.2016.11.112 27915065

[10] Kwon HG, Jang SH. Cingulum injury by external ventricular drainage procedure: diffusion tensor tractography study. Clinical neuroradiology. 2015;25(1):65–7. 10.1007/s00062-013-0269-z 24221532

[11] Horn A, Ostwald D, Reisert M, Blankenburg F. The structural-functional connectome and the default mode network of the human brain. NeuroImage. 2014;102 Pt 1:142–51. 10.1016/j.neuroimage.2013.09.069 24099851

[12] Raichle ME, MacLeod AM, Snyder AZ, Powers WJ, Gusnard DA, Shulman GL. A default mode of brain function. Proceedings of the National Academy of Sciences of the United States of America. 2001;98(2):676–82. 10.1073/pnas.98.2.676 11209064 PMC14647

[13] Reess TJ, Rus OG, Schmidt R, de Reus MA, Zaudig M, Wagner G, et al Connectomics-based structural network alterations in obsessive-compulsive disorder. Translational psychiatry. 2016;6(9):e882 10.1038/tp.2016.163 27598966 PMC5048203

[14] van den Heuvel MP, Bullmore ET, Sporns O. Comparative Connectomics. Trends in Cognitive Sciences. 2016; 20(5), 345–361. 10.1016/j.tics.2016.03.001 27026480

[15] Sporns O, Tononi G, Kötter R. The human connectome: A structural description of the human brain. PLoS computational biology. 2005;1(4):e42 10.1371/journal.pcbi.0010042 16201007 PMC1239902

[16] Toga AW, Clark KA, Thompson PM, Shattuck DW, Van Horn JD. Mapping the human connectome. Neurosurgery. 2012;71(1):1–5. 10.1227/NEU.0b013e318258e9ff 22705717 PMC3555558

[17] Menon V. Large-scale brain networks and psychopathology: a unifying triple network model. Trends in cognitive sciences. 2011;15(10):483–506. 10.1016/j.tics.2011.08.003 21908230

[18] Andersson JLR, Sotiropoulos SN. An integrated approach to correction for off-resonance effects and subject movement in diffusion MR imaging. NeuroImage. 2016;125:1063–1078. 10.1016/j.neuroimage.2015.10.019 26481672 PMC4692656

[19] Vu ATT, Auerbach E, Lenglet C, Moeller S, Sotiropoulos SNN, Jbabdi S, et al High resolution whole brain diffusion imaging at 7T for the Human Connectome Project. NeuroImage. 2015;122:318–31. 10.1016/j.neuroimage.2015.08.004 26260428 PMC4618066

[20] Sotiropoulos SN, Hernández-Fernández M, Vu AT, Andersson JL, Moeller S, Yacoub E, et al Fusion in diffusion MRI for improved fibre orientation estimation: An application to the 3T and 7T data of the Human Connectome Project. NeuroImage. 2016;134:396–409. 10.1016/j.neuroimage.2016.04.014 27071694 PMC6318224

[21] Van Essen DC, Ugurbil K, Auerbach E, Barch D, Behrens TEJ, Bucholz R, et al The Human Connectome Project: a data acquisition perspective. NeuroImage. 2012; 62(4), 2222–2231. 10.1016/j.neuroimage.2012.02.018 22366334 PMC3606888

[22] Fischl B. FreeSurfer. NeuroImage. 2012; 62(2), 774–781. 10.1016/j.neuroimage.2012.01.021 22248573 PMC3685476

[23] Grandhi R, Okonkwo DO. Perioperative Management of Severe Traumatic Brain Injury in Adults. Schmidek and Sweet Operative Neurosurgical Techniques: Indications, Methods, and Results: Sixth Edition. 2012;2:1495–1512. 10.1016/B978-1-4160-6839-6.10132-7

[24] Van Rossum G, Drake FL. The Python language reference manual: for Python version 3.2. Network Theory Ltd; 2011. Available from: https://ir.cwi.nl/pub/5008/05008D.pdf.

[25] Tournier JD, Calamante F, Connelly A. MRtrix: Diffusion tractography in crossing fiber regions. International Journal of Imaging Systems and Technology. 2012;22(1):53–66. 10.1002/ima.22005

[26] Tournier JD, Calamante F, Connelly A. Improved probabilistic streamlines tractography by 2 nd order integration over fibre orientation distributions. Ismrm. 2010;88(2003):2010.

[27] Fischl B, van der Kouwe A, Destrieux C, Halgren E, Ségonne F, Salat DH, et al Automatically parcellating the human cerebral cortex. Cerebral cortex (New York, NY: 1991). 2004;14(1):11–22. 10.1093/cercor/bhg087 14654453

[28] Calamante F, Tournier J, Jackson GD, Connelly A. Track-density imaging (TDI): Super-resolution white matter imaging using whole-brain track-density mapping. Neuroimage. 2010;53(4):1233–43. 10.1016/j.neuroimage.2010.07.024 20643215

[29] Calamante F. Track-weighted imaging methods: extracting information from a streamlines tractogram. MAGMA. 2017 8;30(4):317–35. http://www.ncbi.nlm.nih.gov/pubmed/28181027 28181027 10.1007/s10334-017-0608-1

[30] Willats L, Raffelt D, Smith RE, Tournier JD, Connelly A, Calamante F. Quantification of track-weighted imaging (TWI): Characterisation of within-subject reproducibility and between-subject variability. Neuroimage. 2014;87:18–31. 10.1016/j.neuroimage.2013.11.016 24246491

[31] Atasoy S, Donnelly I, Pearson J. Human brain networks function in connectome-specific harmonic waves. Nature communications. 2016;7:10340 10.1038/ncomms10340 26792267 PMC4735826

[32] Collin G, Scholtens LH, Kahn RS, Hillegers MHJ, van den Heuvel MP. Affected Anatomical Rich Club and Structural-Functional Coupling in Young Offspring of Schizophrenia and Bipolar Disorder Patients. Biological psychiatry. 2017;82(10):746–755. 10.1016/j.biopsych.2017.06.013 28734460

[33] Jones DK, Cercignani M. Twenty-five pitfalls in the analysis of diffusion MRI data. NMR in biomedicine. 2010;23(7):803–20. 10.1002/nbm.1543 20886566

[34] Hagmann P, Cammoun L, Gigandet X, Meuli R, Honey CJ, Wedeen VJ, et al Mapping the structural core of human cerebral cortex. PLoS biology. 2008;6(7):e159 10.1371/journal.pbio.0060159 18597554 PMC2443193

[35] Honey CJ, Sporns O, Cammoun L, Gigandet X, Thiran JP, Meuli R, et al Predicting human resting-state functional connectivity from structural connectivity. Proceedings of the National Academy of Sciences of the United States of America. 2009;106(6):2035–40. 10.1073/pnas.0811168106 19188601 PMC2634800

[36] Achard S, Bullmore E. Efficiency and cost of economical brain functional networks. PLoS computational biology. 2007;3(2):e17 10.1371/journal.pcbi.0030017 17274684 PMC1794324

[37] Zalesky A, Fornito A, Bullmore ET. Network-based statistic: identifying differences in brain networks. NeuroImage. 2010;53(4):1197–207. 10.1016/j.neuroimage.2010.06.041 20600983

[38] Civier O, Smith RE, Yeh CH., Connelly A, Calamante F. Is removal of weak connections necessary for graph-theoretical analysis of dense weighted structural connectomes from diffusion MRI? NeuroImage. 2019; 194(March): 68–81. 10.1016/j.neuroimage.2019.02.039 30844506

[39] Uğurbil K, Xu J, Auerbach EJ, Moeller S, Vu AT, Duarte-Carvajalino JM, et al Pushing spatial and temporal resolution for functional and diffusion MRI in the Human Connectome Project. NeuroImage. 2013;80:80–104. 10.1016/j.neuroimage.2013.05.012 23702417 PMC3740184

[40] Spreng RN. The Fallacy of a “Task-Negative”Network. Frontiers in Psychology. 2012;3(MAY):1–5. 22593750 10.3389/fpsyg.2012.00145 PMC3349953

[41] Sethi A, Sarkar S, Dell’Acqua F, Viding E, Catani M, Murphy DGM, et al Anatomy of the dorsal default-mode network in conduct disorder: Association with callous-unemotional traits. Developmental cognitive neuroscience. 2018;30:87–92. 10.1016/j.dcn.2018.01.004 29413533 PMC5945604

[42] Brown CA, Jiang Y, Smith CD, Gold BT. Age and Alzheimer’s pathology disrupt default mode network functioning via alterations in white matter microstructure but not hyperintensities. Cortex; a journal devoted to the study of the nervous system and behavior. 2018;104:58–74. 10.1016/j.cortex.2018.04.006 29758374 PMC6008234

[43] Lin P, Yang Y, Gao J, De Pisapia N, Ge S, Wang X, et al Dynamic Default Mode Network across Different Brain States. Scientific reports. 2017;7(March):46088 10.1038/srep46088 28382944 PMC5382672

[44] Buckner RL, Andrews-Hanna JR., Schacter DL. Brain’s Default Network. Annals of the New York Academy of Sciences. 2008; 1124(1); 1–38. 10.1196/annals.1440.011 18400922

[45] Whitton AE, Webb CA, Dillon DG, Kayser J, Rutherford A, Goer F, et al Pretreatment Rostral Anterior Cingulate Cortex Connectivity With Salience Network Predicts Depression Recovery: Findings From the EMBARC Randomized Clinical Trial. Biol Psychiatry. 2019;85(10):872–80. 10.1016/j.biopsych.2018.12.007 30718038 PMC6499696

[46] Menon V, Uddin LQ. Saliency, switching, attention and control: a network model of insula function. Brain Struct Funct. 2010 6;214(5–6):655–67. http://www.ncbi.nlm.nih.gov/pubmed/20512370 20512370 10.1007/s00429-010-0262-0 PMC2899886

[47] Sadaghiani S, D’Esposito M. Functional characterization of the cingulo-opercular network in the maintenance of tonic alertness. Cereb Cortex. 2015;25(9):2763–73. 10.1093/cercor/bhu072 24770711 PMC4537431

[48] Glasser MF, Coalson TS, Robinson EC, Hacker CD, Harwell J, Yacoub E, et al A multi-modal parcellation of human cerebral cortex. Nature. 2016;536(7615):171–178. 10.1038/nature18933 27437579 PMC4990127

[49] Kong XZ, Mathias SR, Guadalupe T, ENIGMA Laterality Working Group, Glahn DC, Franke B, et al Mapping cortical brain asymmetry in 17,141 healthy individuals worldwide via the ENIGMA Consortium. Proceedings of the National Academy of Sciences of the United States of America. 2018;115(22):E5154–E5163. 10.1073/pnas.1718418115 29764998 PMC5984496

[50] Sotiropoulos SN, Zalesky A. Building connectomes using diffusion MRI: why, how and but. NMR in Biomedicine. 2017; p. e3752 10.1002/nbm.3752 28654718 PMC6491971

[51] Bassett DS, Brown JA, Deshpande V, Carlson JM, Grafton ST. Conserved and variable architecture of human white matter connectivity. NeuroImage. 2011;54(2):1262–79. 10.1016/j.neuroimage.2010.09.006 20850551

[52] Thomas C, Ye FQ, Irfanoglu MO, Modi P, Saleem KS, Leopold DA, et al Anatomical accuracy of brain connections derived from diffusion MRI tractography is inherently limited. Proceedings of the National Academy of Sciences of the United States of America. 2014;111(46):16574–9. 10.1073/pnas.1405672111 25368179 PMC4246325

[53] Dauguet J, Peled S, Berezovskii V, Delzescaux T, Warfield SK, Born R, et al Comparison of fiber tracts derived from in-vivo DTI tractography with 3D histological neural tract tracer reconstruction on a macaque brain. NeuroImage. 2007;37(2):530–8. 10.1016/j.neuroimage.2007.04.067 17604650

[54] O’Leary ST, Kole MK, Hoover Da, Hysell SE, Thomas A, Shaffrey CI. Efficacy of the Ghajar Guide revisited: a prospective study. Journal of neurosurgery. 2000;92(5):801–803. 10.3171/jns.2000.92.5.0801 10794294

